# Supplementary material for: Effect of Salmonella Typhimurium Colonization on Microbiota Maturation and Blood Leukocyte Populations in Broiler Chickens
Source: Animals (Basel). 2022 Oct 20;12(20):2867. doi: 10.3390/ani12202867 (PMC9597776; doi:10.3390/ani12202867)
Supplement: Supplementary file 1 [file animals-12-02867-s001.zip › animals-1972333-supplementary.pdf]

Table S1: Relative abundance of the top 20 genera following ST colonization.

Genus	D8		D14			D21		
	Control	SA	Control	SA	SN	Control	SA	SN
Clostridiales_unclassified	15.70	13.80	15.61	11.52	8.76	19.73	20.80	18.31
Oscillospira	21.65	11.76	13.40	8.37	13.08	14.22	11.74	12.19
Ruminococcaceae_unclassified	11.09	10.23	15.70	15.39	14.18	15.54	10.53	9.75
Butyricicoccus	3.40	5.47	4.03	9.86	11.96	6.03	10.95	7.61
Lachnospiraceae_unclassified	7.92	8.56	2.93	7.45	1.55	5.60	13.00	10.88
Subdoligranulum	14.63	8.52	0.27	14.01	0.11	0.43	7.55	1.88
Bacteroides	0.00	2.43	0.00	10.39	11.67	0.01	7.39	13.79
Lactobacillus	4.51	22.04	11.67	0.53	0.24	1.92	1.84	0.68
Coprobacillus	3.79	5.25	6.12	2.53	5.59	3.40	3.78	5.83
Enterococcus	3.04	1.45	4.52	2.98	9.08	6.56	2.01	0.90
Escherichia	2.05	0.95	3.32	1.74	2.43	8.44	2.58	4.47
Bacillaceae_unclassified	0.77	3.16	9.93	6.83	0.95	1.47	1.28	0.88
Erysipelatoclostridium	3.73	5.80	1.78	1.57	3.95	3.64	1.46	1.62
cc_115	2.26	1.78	3.60	4.26	4.13	1.51	1.95	0.62
Ruminococcus	1.44	0.60	2.58	0.81	2.82	2.60	2.50	3.12
Dorea	0.12	0.39	0.79	0.53	7.31	2.77	1.20	2.58
Erysipelotrichaceae_unclassified	1.91	1.69	2.74	1.70	2.87	1.57	1.45	1.74
Turicibacter	0.04	0.00	2.86	0.01	0.09	2.72	0.02	0.07
RF39_unclassified	0.74	0.10	0.38	0.49	2.00	0.35	0.34	0.84
Enterobacteriaceae_unclassified	0.51	0.01	0.11	0.75	0.07	0.00	0.68	0.91
Anaeroplasma	0.00	0.00	0.68	0.00	0.37	0.35	0.34	0.91
Christensenellaceae_unclassified	0.50	0.09	0.30	0.07	0.17	0.48	0.10	0.26
Coprococcus	0.24	0.15	0.20	0.04	0.36	0.13	0.48	0.33
Clostridium	0.02	0.12	0.07	0.05	0.40	0.12	0.21	0.12
Mogibacteriaceae_unclassified	0.08	0.00	0.21	0.09	0.13	0.19	0.14	0.21
Lachnospira	0.09	0.06	0.17	0.13	0.24	0.13	0.03	0.12
ML615J-28_unclassified	0.00	0.00	0.83	0.00	0.00	0.00	0.00	0.00
Dehalobacteriaceae_unclassified	0.00	0.00	0.07	0.00	0.03	0.12	0.35	0.05
Blautia	0.16	0.00	0.01	0.06	0.12	0.04	0.05	0.11
Proteus	0.00	0.16	0.02	0.10	0.03	0.03	0.06	0.09
rc4-4	0.00	0.00	0.00	0.00	0.00	0.48	0.00	0.00
Enterobacter	0.00	0.00	0.00	0.00	0.11	0.00	0.22	0.06
Faecalibacterium	0.00	0.02	0.07	0.07	0.04	0.07	0.04	0.05
Holdemania	0.05	0.00	0.04	0.00	0.08	0.02	0.09	0.06
Pediococcus	0.05	0.02	0.11	0.00	0.10	0.00	0.00	0.00
[Eubacterium]	0.02	0.00	0.19	0.00	0.04	0.00	0.00	0.00
Klebsiella	0.00	0.02	0.03	0.04	0.03	0.01	0.00	0.04
Salmonella	0.00	0.11	0.00	0.04	0.00	0.00	0.00	0.00
[Ruminococcus]	0.00	0.02	0.00	0.01	0.00	0.04	0.05	0.00
SHA-98_unclassified	0.00	0.00	0.06	0.00	0.00	0.06	0.00	0.00
Bacteria_unclassified	0.00	0.00	0.00	0.06	0.03	0.00	0.01	0.00
Peptostreptococcaceae_unclassified	0.00	0.00	0.06	0.00	0.00	0.01	0.02	0.00
Shigella	0.03	0.00	0.04	0.00	0.00	0.00	0.00	0.00
EtOH8_unclassified	0.00	0.00	0.00	0.00	0.00	0.07	0.00	0.00

Weissella	0.01	0.02	0.03	0.01	0.00	0.00	0.00	0.00
Clostridiaceae_unclassified	0.00	0.00	0.02	0.00	0.04	0.00	0.00	0.00
Firmicutes_unclassified	0.00	0.00	0.04	0.00	0.00	0.00	0.00	0.01
Staphylococcus	0.00	0.00	0.01	0.01	0.00	0.01	0.00	0.00
Enterococcaceae_unclassified	0.00	0.00	0.00	0.00	0.00	0.02	0.00	0.00

*Data is shown as percent relative abundance of normalized data.
